# Implications of Contact Days in the Treatment of Relapsed Refractory Diffuse Large B-Cell Lymphoma

**DOI:** 10.1093/oncolo/oyad204

**Published:** 2023-07-11

**Authors:** Leyla Bojanini, Neel Gupta, Ali Raza Khaki

**Affiliations:** Division of Oncology, Department of Medicine, Stanford University, Stanford, CA, USA; Division of Oncology, Department of Medicine, Stanford University, Stanford, CA, USA; Division of Oncology, Department of Medicine, Stanford University, Stanford, CA, USA

## Abstract

This commentary remarks on a recently published study assessing clinic contact days for patients enrolled in the Canadian Cancer Trials Group LY.12 clinical trial.

Diffuse large B-cell lymphoma (DLBCL) is the most common subtype of non-Hodgkin lymphoma (NHL), with an incidence of 5.5 cases per 100 000 persons per year in the US.^1^ In the immunochemotherapy era, more than 50% of patients are cured with front-line cyclophosphamide, doxorubicin, vincristine, and prednisone (R-CHOP). However, 20%-50% of patients will either be refractory to R-CHOP or relapse shortly after experiencing a complete response (CR) to front-line therapy.^2^ For patients with relapsed/refractory (R/R) DLBCL, salvage chemotherapies followed by autologous stem-cell transplant (ASCT) or chimeric antigen T-cell (CAR-T) therapies have all been used to further increase fraction cured of this aggressive lymphoma. However, these therapies have associated toxicities as well as cost and time requirement that may significantly impact patients.

In this issue of *The Oncologist*, Gupta et al. report an exploratory analysis assessing clinic contact days for patients enrolled in the Canadian Cancer Trials Group LY.12 clinical trial involving patients with R/R DLBCL.^3^ Prior to this study, the authors have highlighted the high time demands (which they have termed “time toxicity”) for patients and care partners while receiving anti-cancer therapy.^4,5^ Their prior work has largely focused on solid tumors, and the current study extends this investigation to high grade lymphoma. Using trial records, they quantify the number of days patients spent with in-person healthcare contact versus the days spent at home between 2 trial arms.

Briefly, CCTG LY.12 trial (ClinicalTrials.gov Identifier: NCT00078949) was a randomized trial published in 2014 that enrolled patients with aggressive NHL histology that had either relapsed or been refractory to first-line treatment. Two different salvage chemotherapy regimens were compared in the study: gemcitabine, dexamethasone, cisplatin (GDP), an outpatient chemotherapy regimen, versus dexamethasone, high dose cytarabine, and cisplatin (DHAP), a regimen that requires inpatient administration. The study found that GDP was noninferior to DHAP in terms of transplantation rate (52% vs. 49%), ORR (45% vs. 44%), event-free survival (HR, 0.99; 95%CI: 0.82 to 1.21; *P* =.95), and overall survival (HR, 1.03; 95% CI, 0.83 to 1.28; *P* =.78). Treatment with GDP was associated with less toxicity and required less hospitalizations for management of adverse events^6^ and has become one of several standard of care salvage chemotherapy regimens prior to ASCT.

In the current analysis, the authors compared differences in patient contact days between the 2 arms of the study and found that despite comparable oncologic outcomes, GDP was associated with significantly less contact time. Contact days were defined as days with in-person healthcare contact and were ascertained by analyzing available trial forms from the time of study assignment to progression or transplant. The days without in-person healthcare contact were named “home days.” Although contact days were comparable in both arms (median: 18 vs. 19, *P* = .79), the GDP arm had more home days (median: 33 vs. 28, *P* < .001) and a lower proportion of contact days (34%, vs. 38%, *P* = .009). As may be expected, the GDP arm contact days were mostly related to planned outpatient chemotherapy (median, 10 vs. 8 days), and the DHAP arm experienced more contact days related to inpatient hospitalization (median: 11 vs. 0 days). The authors conclude that such information may help inform patients and physicians with respect to choice of salvage regimen.^3^

We commend the authors on this analysis as well as their prior work on defining and contextualizing the time cost associated with cancer therapy. However, it is worth exploring whether this metric is similarly applicable to patients with R/R lymphoma as represented in the CCTG LY.12 trial. For example, patients requiring salvage chemotherapy such as DHAP or GDP have relapsed or refractory disease that will subsequently require ASCT or CAR-T therapy depending on response to these salvage regimes. These postsalvage therapies are relatively toxic, resource-, and time-intensive therapies that require close monitoring. As such, any potential advantage in number of contact days gained during the salvage chemotherapy stage of treatment would likely be obscured by the necessary contact days associated with subsequent transplant or CAR-T therapy. Moreover, we would posit that given curative intent with these maneuvers, of far greater importance is the expected efficacy as a function of prior lines of therapy (ie, choosing non-cross resistant agents) and/or toxicity profile. This is in distinction to the differences highlighted in their work in solid organ tumor patients. In that population, ­palliative, systemic therapy might be expected to extend survival by several months, highlighting the importance of measuring and minimizing contact time.

That said, contact time is not wholly irrelevant in the treatment of patients with hematologic malignancies. Specific settings within which time toxicity should be further explored include but are not limited to patients receiving cellular therapies in the form of bispecific antibodies (almost all have indefinite schedules, though some are required to start inpatient for extended periods of time) or various CAR T-cell products that can be administered either in the hospital or in monitored outpatient settings. In addition, while oral medications for various lymphomas are thought of as less burdensome alternatives to intravenous therapy, many regimens require extended hospitalizations during their dose-escalation phase to manage potential toxicities such as tumor lysis syndrome or severe cytopenias.

One other notable distinction in this study as compared to prior work from the authors was the description of time spent with clinic contact as “contact days” rather than “time toxicity.” We agree with this decision, as it more appropriately acknowledges that time spent in healthcare may be perceived differently depending on the circumstances of the patient and care partner and thus can be difficult to establish whether healthcare contact is in fact “toxic” from a patient’s perspective. One could hypothesize the potential benefits of time spent in healthcare-related activities such as a sense of security, timely symptom control, increased assistance for patients that lack an adequate support network, shelter for the homeless, respite for patients and/or care partner, etc. Conversely, a study by Hall and colleagues involving 29 participants (11 patients, 7 caregivers, and 11 oncology professionals) at an academic institution revealed that patients and caregivers often expressed feeling stressed and burdened by the time required to receive cancer care. The study found that discussions of time required during cancer care were often prompted by isolated incidents such as long waiting times at the lab center or clinic, long commute times, etc. Patients and caregivers often reported exhaustion that contributed to feelings of distress. Moreover, patients and caregivers described feelings of regret over losing the ability to travel or do other leisure activities.^7^ This study, along with the work done by Gupta and colleagues, highlights the importance of having a clear understanding of the time required for cancer-related care, and how this can aid in setting expectations and creating a “road map” of a realistic time estimate for proposed treatments.

Further qualitative work is required to understand patients’ perceptions about the true “toxicity” of time spent in healthcare-related activities, and how these perceptions may differ depending on patient’s socioeconomic or racial/ethnic background. Moreover, given recent transition to the use of telemedicine practices as a result of the COVID 19 pandemic, making the distinction between telehealth care contact versus in-person healthcare contact and how these impact the time burden on patients and care partner is crucial to translate the results of qualitative work as close to what it would be like in the real-world setting. This is especially important given a recent study showing that among the 33 318 patients with cancer interviewed, those who completed telemedicine visits reported consistently better experience of care compared to those who completed in-person visits. Specifically, the telemedicine group reported high patient experience scores in access-related questions compared with the in-person group.^8^

Guiding patients and families in making decisions that are goal concordant is a key component of physician-patient communication. We congratulate Gupta et al for their important work measuring contact days and time toxicity for patients with cancer. Further work is needed to accurately quantify healthcare contact days for different cancer treatments and contextualize within the goals of patients and how these offset potential life extension offered by respective treatments.
